# Efficacy of artesunate-amodiaquine for treating uncomplicated falciparum malaria in sub-Saharan Africa: a multi-centre analysis

**DOI:** 10.1186/1475-2875-8-203

**Published:** 2009-08-23

**Authors:** Julien Zwang, Piero Olliaro, Hubert Barennes, Maryline Bonnet, Philippe Brasseur, Hasifa Bukirwa, Sandra Cohuet, Umberto D'Alessandro, Abdulaye Djimdé, Corine Karema, Jean-Paul Guthmann, Sally Hamour, Jean-Louis Ndiaye, Andreas Mårtensson, Claude Rwagacondo, Issaka Sagara, Albert Same-Ekobo, Sodiomon B Sirima, Ingrid van den Broek, Adoke Yeka, Walter RJ Taylor, Grant Dorsey, Milijaona Randrianarivelojosia

**Affiliations:** 1 Shoklo Malaria Research Unit (SMRU), Mae Sot, Thailand; 2 UNICEF/UNDP/WB/WHO Special Programme for Research and Training in Tropical Diseases, Geneva, Switzerland; 3 Institut de la Francophonie pour la Médecine Tropicale, BP 9519, Vientiane, Lao PDR; 4 Epicentre, Paris, France; 5 Institut de Recherche pour le Développement (IRD), Dakar, Sénégal; 6 Uganda Malaria Surveillance Project, Kampala, Uganda; 7 Department of Parasitology, Institute of Tropical Medicine, Antwerp, Belgium; 8 Malaria Research and Training Center, Department of Epidemiology of Parasitic Diseases, Faculty of Medicine and Pharmacy, University of Bamako, Bamako, Mali; 9 National Malaria Control Programme, Kigali, Rwanda; 10 Department of Parasitology, Faculty of Medicine, Cheikh Anta Diop University, Dakar, Senegal; 11 Infectious Diseases Unit, Department of Medicine, Karolinska University Hospital, Karolinska Institute, Stockholm, Sweden; 12 Laboratoire de Parasitologie, Centre Hospitalier Universitaire, Yaoundé, Cameroun; 13 Centre National de Recherche et de Formation sur le Paludisme, Ministère de la Santé, Ouagadougou, Burkina Faso; 14 Médecins sans Frontières, London, UK; 15 Department of Medicine, University of California San Francisco, San Francisco, California, USA; 16 Unité de Recherche sur le Paludisme, Institut Pasteur, Antananarivo, Madagascar

## Abstract

**Background:**

Artesunate and amodiaquine (AS&AQ) is at present the world's second most widely used artemisinin-based combination therapy (ACT). It was necessary to evaluate the efficacy of ACT, recently adopted by the World Health Organization (WHO) and deployed over 80 countries, in order to make an evidence-based drug policy.

**Methods:**

An individual patient data (IPD) analysis was conducted on efficacy outcomes in 26 clinical studies in sub-Saharan Africa using the WHO protocol with similar primary and secondary endpoints.

**Results:**

A total of 11,700 patients (75% under 5 years old), from 33 different sites in 16 countries were followed for 28 days. Loss to follow-up was 4.9% (575/11,700). AS&AQ was given to 5,897 patients. Of these, 82% (4,826/5,897) were included in randomized comparative trials with polymerase chain reaction (PCR) genotyping results and compared to 5,413 patients (half receiving an ACT).

AS&AQ and other ACT comparators resulted in rapid clearance of fever and parasitaemia, superior to non-ACT. Using survival analysis on a modified intent-to-treat population, the Day 28 PCR-adjusted efficacy of AS&AQ was greater than 90% (the WHO cut-off) in 11/16 countries. In randomized comparative trials (n = 22), the crude efficacy of AS&AQ was 75.9% (95% CI 74.6–77.1) and the PCR-adjusted efficacy was 93.9% (95% CI 93.2–94.5). The risk (weighted by site) of failure PCR-adjusted of AS&AQ was significantly inferior to non-ACT, superior to dihydroartemisinin-piperaquine (DP, in one Ugandan site), and not different from AS+SP or AL (artemether-lumefantrine). The risk of gametocyte appearance and the carriage rate of AS&AQ was only greater in one Ugandan site compared to AL and DP, and lower compared to non-ACT (p = 0.001, for all comparisons). Anaemia recovery was not different than comparator groups, except in one site in Rwanda where the patients in the DP group had a slower recovery.

**Conclusion:**

AS&AQ compares well to other treatments and meets the WHO efficacy criteria for use against falciparum malaria in many, but not all, the sub-Saharan African countries where it was studied. Efficacy varies between and within countries. An IPD analysis can inform general and local treatment policies. Ongoing monitoring evaluation is required.

## Background

Artemisinin-based combination therapy (ACT) is now the treatment of choice for uncomplicated *Plasmodium falciparum *malaria. As of February 2009, more than 80 countries worldwide have adopted ACT as first-line therapy [1]. Currently, four forms of ACT are recommended by the World Health Organization (WHO): artemether and lumefantrine (AL), artesunate and amodiaquine (AS&AQ), artesunate and mefloquine (AS+MQ) and artesunate and sulphadoxine-pyrimethamine (AS+SP) [2]. The choice of ACT for a country or a region depends on a number of considerations. A critical element is the level of underlying resistance to the longer-acting partner drug in the combination. This is particularly important for amodiaquine (AQ) and sulphadoxine-pyrimethamine (SP) in Africa, where both drugs have been widely used as monotherapies.

The WHO recommends that countries use ACT, which is at least 90% effective and introduce new forms of ACT that are at least 95% effective after discounting reinfections (PCR-adjusted) and that the Day 28 efficacy of respective partner drugs alone should exceed 80% [2]. Concerns have been raised over ACT including amodiaquine (AQ) meeting such criteria in areas where AQ has been widely used as monotherapy. The second most widely used ACT, AS&AQ has been adopted as first-line treatment in 18 countries in Africa (Burundi, Cameroon, Chad, Congo, Côte d'Ivoire, Democratic Republic of Congo, Equatorial Guinea, Gabon, Ghana, Guinea, Liberia, Madagascar, Mauritania, Sénégal, Sao Tomé & Principe, Sierra Leone, Sudan [South], Zanzibar) and Indonesia. The AS&AQ combinations have been available in a non-fixed formulation (AS+AQ) as either a loose combination or as blister-packed tablets from several pharmaceutical companies, and more recently, as a fixed-dose drug combination (ASAQ). Therefore, the widespread use of these combinations calls for a comprehensive synthesis of published and unpublished available results to properly inform policy decisions.

The efficacy and tolerability of AS&AQ has been tested formally in several clinical trials in different epidemiological settings in Africa. Following a systematic review of published and unpublished comparative and non-comparative trials (Olliaro *et al*, personal communication), investigators were contacted for individual patient data. This resulted in a pooled multi-centred analysis ([3-24]; Bonnet *et al*, unpublished data, 2004; van den Broek, unpublished data, 2005; Cohuet *et al*, unpublished data, 2004; Grandesso, unpublished data, 2004), which cannot be strictly defined as a meta-analysis since it does not include exhaustively all the trials with AS&AQ, an element that may introduce a selection bias. However, to date, this analysis with 26 trials enrolling 11,700 patients mostly in randomized comparative trials with genotyping, is the largest analysis at the individual patient level ever compiled for an ACT. In addition to examining the primary parasitological outcomes, this study also analysed the resolution of parasitaemia, fever, gametocytaemia and anaemia, and modelled risk factors for treatment failure.

## Methods

### Study sites, design and patients

The studies were identified through a systematic review of comparative and non-comparative clinical trials conducted in sub-Saharan Africa, using any formulation of AS&AQ for treating uncomplicated falciparum malaria with follow-up of at least 28 days, regardless of language or publication status (published, unpublished, in press, technical reports) (Olliaro *et al*, personal communication).

Published studies were identified through electronic searches up to April 2007 of MEDLINE, EMBASE, LILACS, the Cochrane Infectious Diseases Group's trials register and the Cochrane Central Register of Controlled Trials (CENTRAL) using the following search terms: malaria, amodiaquine, artesunate and artemisinins. Unpublished studies were identified through personal contacts and by manually searching the reference lists of studies identified by the above-mentioned methods, contacting individual researchers working in the field, and examining WHO records. For all studies identified, the corresponding author was contacted and asked to provide individual patient data.

The following aspects of methodological quality of the received data sets/publications were assessed: generation of allocation sequence, adequacy of concealment of the allocation of treatment, degree of blinding, and completion of follow-up. Generation of the allocation sequence and allocation concealment were classified as adequate, inadequate, or unclear [25]. Blinding was classified as open, single, or double. Losses to follow-up (regardless of reasons) were computed and considered adequate if less than 10%. Finally, based on the presented power calculation, sample size estimation was assessed, as was whether an intention-to-treat analysis could be computed. The last patient included in this analysis was enrolled in December 2006. Studies involving pregnant women or severe malaria, studies performed outside sub-Saharan Africa, as well as economic and pharmacokinetic analyses, were excluded.

**Figure 1:**
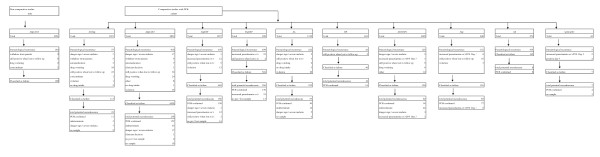
**Flow chart of comparative and non comparative studies by anti-malarial drug**.

A total of 46 trials were identified, of which 42 compared AS&AQ with other anti-malarial drugs; four were non-comparative. Seven studies were excluded, four because follow-up was limited to 14 days [26-29], and three because they were not in Africa (Afghanistan, Colombia, Indonesia) [30-32]. Of the remaining, 39 identified studies fulfilling the inclusion criteria, 14 had to be excluded because individual patient data were not made available. The remaining 25 studies, and a randomized comparative trial conducted in a common site in Uganda-Apac [24] comprised a total of 11,700 patients treated with either a non-fixed AS+AQ (N = 4,914) or fixed ASAQ (N = 1,073). In randomized comparative trials the AS&AQ groups (82% N = 4,826/5,897) were compared to anti-malarial drugs: AQ (N = 648), AS (N = 279), AS+SP (N = 1,005), AQ+SP (N = 1,257), AL (N = 1,319), dihydroartemisinin-piperaquine (DP, N = 463), chloroquine + SP (CQ+SP, N = 699), or quinine + chloroquine (Q+CQ, N = 43) (Figure 1).

### Treatments

AS&AQ treatment regimens. AS&AQ products were either loose or co-blister-packed combinations, individually formulated products (AS+AQ), or fixed-dose co-formulations (ASAQ). In general, the loose AS+AQ were dosed based on body weight, while in a few studies the co-blister-packed AS+AQ and the co-formulated ASAQ were based on age and weight range.

The majority (82%, 4,914/5,987) of the patients were treated with individually formulated AS and AQ. The target dose was AS 12 mg/kg over 3 days and AQ 30 mg/kg over 3 days, except in Uganda where AQ was given at 25 mg/kg (10 mg/kg on Days 0 and 1 then 5 mg/kg on Day 2). The co-blister-packed AS+AQ (AS 50 mg + AQ 153 mg base for each of the days of treatment, dose ratio = 3.1) was used in two studies in Senegal [19,23] containing for each of the days of treatment 1 tablet of AS 50 mg and 1 of AQ 153 mg (base), dosed either by age or by weight. When used by age, the dosing categories were: (i) <1 y: 1/2 tablet; (ii) ≥ 1 to 6 y: 1 tablet; (iii) ≥ 7 to <13 y: 2 tablets; (iv) ≥ 13 y: 4 tablets. The fixed-dose combinations of ASAQ were available as two-and three-tablet strength products given by age. The fixed-dose combination was also given either once or twice a day [4,5]. For the two-tablet strength fixed-dose combination ASAQ (paediatric AS 25 mg + AQ 67.5; adult AS 100 mg + AQ 270 mg, dose ratio = 2.7), the dosing categories [33-35] were: (i) 0–1 months: 1/2 paediatric; (ii) 2–11 months: 1 paediatric; (iii) 1–6 years: 2 paediatric; (iv) 7–13 years: 1 adult; and (v) ≥ 14 years: 2 adult. For the three-tablet strength ASAQ, age- and weight-based doses were administered once-a-day for three days: one tablet/day for children up to 13 years of age (≤ 35 kg) or two tablets/day for adolescents aged 14 years and above and adults (≥ 36 kg). Doses available were: infants (2 to 11 months) received AQ 25 mg/AS 67.5 mg; young children (1 to 4 years) received AQ 50 mg/AS 135 mg; children (6 to 13 years) received 1 tablet/day of AQ 100 mg/AS 270 mg, and adults (14 years or more) received two tablets (AQ 100 mg/AS 270 mg) per day [4].

#### Comparator treatment regimens

(i) for the ACT groups: AL (20 mg artemether/120 mg lumefantrine given according to weight as 1 [5–14 kg], 2 [15–24 kg], 3 [25–34 kg], and 4 [≥ 35 kg] tablets given twice daily for 3 days); DP (around 2.3 mg/kg/day dihydroartemisinin and 18.4 mg/kg for 3 days); AS+SP (AS 4 mg/kg/day; SP 25 mg/kg of sulphadoxine and 1.25 mg/kg/of pyrimethamine administered in a co-formulated tablet [SP] as a single dose)

(ii) for the non-ACT groups: AQ+SP (AQ 10 mg/kg/day for 3 days and SP 25 mg/kg of sulphadoxine and 1.25 mg/kg/of pyrimethamine administered in a co-formulated tablet [SP] as a single dose); CQ (25 mg/kg chloroquine over 3 days) and SP; AQ only (10 mg/kg/day for 3 days); AS5 only (AS 12 mg/kg over 5 days).

### Study endpoints and statistical analysis

The primary endpoint was treatment efficacy by Day 28 defined prospectively in all studies as the treated population free of failure (PCR not adjusted: recurrence, or PCR confirmed: recrudescence). Data were standardized in order to be pooled and to allow for a modified intention-to-treat analysis (mITT). Patients lost to follow-up (or missing a weekly visit) or with any *P. falciparum *infection during the follow-up were censored for the primary outcome at the time they were last seen. Efficacy was measured using Kaplan-Meier survival analysis.

Patients were censored as non-failures when last seen if: (a) lost to follow-up with no evidence of failure, (b) having withdrawn consent, (c) taking a drug with anti-malarial activity, (c) having another illness or being a protocol violation, or (e) having never started the assigned treatment.

A treatment failure was defined as any of the following: (i) the development of danger signs or severe malaria or death or drug-induced vomiting requiring rescue treatment (ii) Day 2 parasitaemia > Day 0 parasitaemia, (iii) Day 3 parasitaemia >25% of the baseline, (iv) Day 7 parasitaemia, and (v) a recurrent parasitaemia within 28 days i.e. a conversion from a positive to a negative smear result sustained to Day 28. Patients who had a treatment failure with missing PCR samples or indeterminant PCR results were classed as recrudescent failures to prevent from overestimating efficacy levels. Treatment failure was considered the sum of early and late treatment failures, as defined by the WHO [2].

The secondary outcomes and analytical method by Day 28:

(i) Parasitaemia clearance times (negative slide)

(ii) Elevated temperature (≥ 37.5°C)

(iii) The risks of recurrence or recrudescence (using PCR) in AS&AQ groups compared to the comparator groups were assessed by Cox regression stratified by site in an attempt to account for potential statistical heterogeneity (assessments made consistently within each study but at different times across studies), and presented as Adjusted Hazard Ratio (AHR).

(iv) The predictors of recurrent and recrudescent infections in AS&AQ groups were similarly assessed by Cox regression stratified by site.

(v) Gametocytaemia was defined as any positive slide for gametocytes, and was analysed as a binary variable. The predictors of patent gametocytaemia on admission were assessed by logistic regression controlled by sites. The overall cumulative incidence probability of gametocyte appearance on Day 28 was defined as the first positive slide during the follow-up being the censoring event and a measured by Kaplan-Meier survival analysis. The carriage rates were calculated in person-week-gametocyte (PGW), compared using the Mantel-Haenszel method to estimate a combined odds ratio between treatments and presented as Rate Ratio (RR_MH_). The risks of gametocyte appearance post-admission were assessed by Cox regression stratified by site.

(vi) Fractional change in haemoglobin or haematocrit between Days 0 and 7 and Days 0 and 28, and anaemia (the cut off was set at Hct <30% or a Hgb <10 mg/L) at baseline and recovery (when HCT became ≥ 30% or Hgb ≥ 10 mg/L) were compared using a student-t paired analysis. Student-t test was used for comparisons between paired mean results. Anaemia defined as <30% Hct or <10 g/dL was categorized into 4 grades [36,37]:

a. grade 1 was ≥ 10 g/dL or ≥ 30% Hct

b. grade 2 was mild (8–9.9 g/dL, or 25%–29.9% Hct)

c. grade 3 was moderate (5–7.9 g/dL, or 15%–24.9% Hct)

d. grade 4 was severe anaemia (<5 g/dL, or <15% Hct)

Parasitaemia, fever, and gametocyte clearance times for each treatment group were compared using the Mann-Whitney test. The proportions of patients remaining febrile on Day 2 or with a positive slide on Day 3 in each treatment group were compared by logistic regression controlled by sites.

For patients who cleared parasites, fever, or anaemia (i, ii, vi respectively), the time of the first negative result (followed by negative counts) was taken as time of clearance. Patients were seen each day for as long as they stayed parasitaemic and thus parasite clearance could be assessed as those who cleared 24, 48, and 72 hours after treatment administration. However given the potential absence of data after treatment (72 hours) for fever and parasite clearance, multivariate analysis was focused on the proportion of patients remaining parasitaemic on Day 3 in comparative trials.

Additionally, any positive gametocyte counts (v) detected any time after treatment start defined gametocytes carriage. Gametocyte carriage rate was expressed in PGW per 1,000 person-weeks followed-up calculated as the total length of gametocyte carriage divided by the total number of persons exposed [38].

Spearman bivariate correlation was noted r_s_. Confidence intervals were calculated at 95%, and comparisons considered significant when P < 0.05. Data were analysed using Stata v10 (Stata Corp.).

### Heterogeneity

Differences in settings, age group, use of PCR, trial design and study procedures were included in the assessment of study heterogeneity using Cochran's Q test and the I^2 ^test [39].

### PCR methods

PCR was performed on paired samples to compare the parasites' genotypes and thus distinguish between new and recrudescent infections. Allelic variation within the merozoite surface protein 1 and 2 (MSP1 and MSP2, respectively), and glutamate reach protein (GLURP) was used in Angola, Congo, DRC, Kenya, Senegal, Sudan, and Uganda [40]. MSP1 and MSP2 were analysed in Angola, Burkina Faso, Gabon, Guinea, Madagascar, Mali, Democratic Republic of Congo (DRC), Rwanda, Sudan, Sierra Leone, and Uganda [41,42]. MSP2 alone was used in Zanzibar and Uganda.

In Uganda, selected regions of the MSP1, MSP2 and 6 microsatellite markers were amplified using PCR and characterized based on sequence and size polymorphisms identified by gel electrophoresis [43].

A recrudescent infection was defined as one that matched in size at least one allele of both the MSP1 and MSP2 loci present in the first sample. Thus, if any clone of a polyclonal primary infection was detected during a second episode, this was considered to be a recrudescence and thus a treatment failure.

### Ethical issues

All the studies had been approved by the relevant ethics and institution review committees.

## Results

### Characteristics of included studies

This review extends from February 1999 in Kenya [39] to December 2006 in Senegal [31] and included only children aged between four and 59 months in half (13/26) of the studies. The proportion of patients lost to follow-up was <10% in all the included trials. A total of 11,700 patients were enrolled in 16 countries at 33 different sites. Individually, the trials enrolled between 27 and 890 ASAQ-treated patients. The total number of patients treated with ASAQ was 5,987, of which 82% (N = 4,896) were enrolled in randomized comparative trials. In comparator arms (N = 5,713), 49% (N = 2,787) were treated with other ACTs and 51% (N = 2,926) treated with non-ACTs (Figure 1). The overall loss to follow-up by Day 28 was 4.9% (575/11,700).

### Study design

Eighteen trials were randomized, comparative, and open label [5-8,10-16,21,22]; Grandesso S, unpublished data, 2004; Cohuet S, unpublished data, 2004; Bonnet M, unpublished data, 2004; van den Broek I, unpublished data, 2005), four were single blinded [3,4,18,31], 1 was placebo-controlled [11], and 3 were non-comparative [9,19,23].

Twenty-two (22) of the 25 studies included applied the Consort guidelines. In the comparative trials, methods of assigning patients to treatments varied from not specified (N = 6), to allocation by bloc (N = 7), age group (N = 3), computer generation (N = 2), sequential alternation (N = 1), slip of paper (N = 1), stratified (N = 1), and variable blocks (N = 1). Treatment allocation was double blinded (N = 1), not reported (N = 3), not specified (N = 8), not done (N = 1), staff blinded (N = 7), and concealed in sealed envelopes (N = 2). In all cases, allocation was not disclosed to investigators at study site until a patient had given written consent to participate in the study. All treatments were supervised over the three-day course. All studies considered followed patients for 28 days or more [10,14,32], but in order to standardize the analysis, only results up to 28 days were included.

In all the included studies, the primary treatment outcome was treatment efficacy. In 22 trials, parasites were genotyped to distinguish new from recrudescent infections (10,077/11,700; 86% of the patients), one of which was not comparative (126/11,700 or 1% of the patients). One study compared non-fixed and fixed combinations of AS&AQ [5]. Comparative studies included 4,896 patients on AS&AQ and 5,713 on other anti-malarials: AL in 11 sites (24%), DP in four sites (8%), AS+SP in nine sites (18%) for ACT groups, and AQ+SP in eight sites (22%), CQ+SP in four sites (12%), AQ in seven sites (11%), AS in two sites (5%), and Q+CQ in one site (1%) (Figure 1).

Day 0 Hgb and Hct levels were available in 87.1% of all the patients (10,944/11,700), 46.9% of whom (N = 4,779) on AS&AQ. The mean change in Hgb or Hct (± SD) could be calculated using a student paired analysis in 2,405 patients (1,361 on AS&AQ), and in 5,388 patients on Day 28 (2,753 on AS&AQ) respectively. All trials recorded gametocyte carriage at study enrolment and during follow-up.

Two studies were multi-centre: (i) a double-blinded comparison of Placebo+AQ to AS+AQ in Senegal, Gabon, and Kenya [11]; and (ii) an open-label comparison of a fixed-dose combination ASAQ vs. AL in Senegal (2 sites), and Cameroon, Madagascar, and Mali (1 site each) [4]. Three (12%, 3/25) unpublished reports from Epicentre (n = 3) were included, and represented 5% of the total patients (639/11,700). One study from Uganda was conducted in four geographical areas with different transmission intensities.

### Demography

The majority of the patients treated with AS&AQ were between six months to five years of age (N = 4,153, 69%, of whom 20%, N = 1,177 were one year old or younger). The five to 14 years olds were 22% (N = 1,307), and adults (15 years or older), 8% (N = 449). The median age was three years, and the range was six months to 89 years. Most of the adults (81%, N = 364) were from Senegal. Uganda contributed the largest percentage of children by country (24%, N = 1,002). In randomized comparative trials, the proportions of children less than five years of age were 81% in both the ASAQ groups (3,918/4,826) and the comparator groups (4,618/5,711).

The minimum patient weight was 5 kg and mean (SD) weight was 10.7 kg (2.8) for young children, 25 kg (8.4) in 5–14 year olds, and 54 kg (10.6) in adults. The proportion of male participants was 53%, ranging from 43% in Sierra Leone to 57% in Senegal.

### Heterogeneity

While all trials had similar endpoints, there were differences in trial design, age group, and PCR genotyping, and substantial heterogeneity was detected due to the inclusion of non-comparative studies and large differences between field sites (I^2 ^test = 83%, p = 0.001, Cochran Q test for heterogeneity). Therefore in an attempt to account for statistical heterogeneity non-comparative studies were not included in most of the analysis and all analysis were stratified by site.

### Primary efficacy outcomes

Of the 5,987 patients treated with AS&AQ, 1% (45/5,987) were censored on Day 0 from the analysis for protocol violation, consent withdrawal, or self-medication as predefined by the mITT analysis. Of the patients treated with AS&AQ, 1,235 were censored by Day 28 for the efficacy analysis (Table 1).

**Table 1:** Censoring events by drug category for the efficacy analysis (both comparative and non-comparative studies included)

		AS&AQ		Other ACTs		non-ACT	
Criteria		n	%	n	%	n	%
**Total**		**5987**	**100%**	**2787**	**100%**	**2926**	**100%**
Censored on Day 0	- no drug intake			3			
	- voluntary withdrawal	19					
	- self-medication	10				16	
	- protocol violation	16		18			13
**Sub-total**		**5942**	**99%**	**2769**	**99%**	**2897**	**99%**
Failure	Total recurrent cases	1126	91%	429	95%	1171	95%
	- PCR confirmed*	203		95		436	
	- indeterminate*	47		47			
	- no PCR/no sample*	20		51		34	
	Danger sign/severe malaria/death*	24	2%	8	2%	1	<1%
	Increased parasitaemia or >25% Day3*					45	3%
	Clinician decision*	1	<1%				
	Drug vomiting	44	4%	6	1%		<1%
	Still positive when lost to follow-up*	29	2%	5	1%	13	1%
	Lost result*	11	1%	3	1%	8	1%
	**Total**	1235	100%	451	100%	1238	100%

* Classified as recrudescent failure

ACT: artemisinin combination therapy.

### Crude Day 28 outcomes (not PCR adjusted)

All studies (comparative and non-comparative). The details of the Kaplan-Meier analysis for a hypothetical cohort of 1,000 patients free of failure are shown in Table 2 for AS&AQ and comparators (ACT and non-ACT).

**Table 2:** Crude efficacy (not PCR-adjusted), number and quotients of failures by Day and drug categories within 28 days*

	artesunate amodiaquine combinations				Other ACT				non-ACT			
	
Day of censure	Failure (n)	Total follow-up	Quotient Day(x)	Free of failure	Failure (n)	Total follow-up	Quotient Day(x)	Free of failure	Failure (n)	Total follow-up	Quotient Day(x)	Free of failure
0	77	5942	0.013	987	8	2769	0.003	997	11	2926	0.004	996
1	24	5839	0.004	983	8	2746	0.003	994	16	2912	0.005	991
2	4	5806	0.001	982	3	2736	0.001	993	22	2895	0.008	983
3	1	5774	0.000	982	1	2729	0.000	993	24	2868	0.008	975
7	5	5745	0.001	981	2	2720	0.001	992	86	2824	0.030	945
14	103	5675	0.018	964	22	2700	0.008	984	228	2703	0.084	866
21	531	5477	0.097	871	147	2653	0.055	929	491	2419	0.203	690
28	490	4874	0.101	783	260	2479	0.105	832	360	1895	0.190	559

Total	1235	5942			451	2769			1238	2926		

*Note: this table does not refer to randomized comparative trials therefore direct comparisons between drug categories in this table are not legitimate. ACT: artemisinin combination therapy.

The overall Day 28 efficacy of AS&AQ was 78.3% (95% CI 77.2–79.4) and 75.9% (95% CI 74.6–77.1) for all trials and comparative trials only, respectively. Efficacies of other forms of ACT were 83.2% (95% CI 81.8–84.6), and for non-ACT 55.8% (95% CI 54.0–57.7)(Table 2). Crude AS&AQ efficacy varied widely across study sites (p = 0.001, log rank test), ranging from 30% (95% CI 25.4–34.5) in Tororo, Uganda, to 100% (95% CI 97.2–100) in Cameroon. The median (range) time to failure in AS&AQ groups was 21 days (0–28). Efficacy by site was positively correlated to median time to failure (r_s _= 0.624, p = 0.001).

### PCR-adjusted Day 28 outcomes

Among AS&AQ recipients, genotyping was available in 21 randomized comparative studies at 23 sites (78% or 4,577/5,987). Of these patients, 257 had a recrudescent infection (Table 3) of which 90% (232/257) were PCR confirmed, 24 were danger signs or severe malaria, and one was resulting from the clinician decision. Overall, 93.9% (95% CI 93.2–94.5) of the patients treated with AS&AQ had cleared their primary infection and were free of recrudescence within 28 days. The corresponding figures were for other forms of ACT 94.8% (95% CI 93.8–95.6) and for non-ACT 80.6% (95% CI 78.8–82.0).

**Table 3:** PCR-adjusted efficacy, number of failures by Day, and drug categories within 28 days (only in studies using PCR results)*

	artesunate amodiaquine combinations				Other ACT				non-ACT			
	
Day of censure	Recrud. (n)	total	Quotient Day(x)	Free of recrud.	Recrud. (n)	total	Quotient Day(x)	Free of recrud.	Recrud. (n)	total	Quotient Day(x)	Free of recrud.
0	18	4577	0.004	996	4	2741	0.001	999		2637	0.000	998
1	6	4559	0.001	995	4	2740	0.001	997	6	2637	0.002	996
2		4549	0.000	995	2	2735	0.001	996	21	2630	0.008	988
3	1	4527	0.000	995	1	2730	0.000	996	20	2604	0.008	979
7	3	4508	0.001	994		2721	0.000	996	35	2565	0.014	966
14	23	4459	0.005	989	10	2701	0.004	992	89	2452	0.036	932
21	119	4292	0.027	961	32	2654	0.012	980	163	2178	0.075	862
28	87	3745	0.025	939	82	2480	0.033	948	109	1685	0.065	806

Total	257	4577			135	2741			443	2637		

*Note: this table does not refer to randomized comparative trials therefore direct comparisons between the groups of this table are not legitimate. ACT: artemisinin combination therapy. Recrud.: recrudescence

The WHO criterion of >90% efficacy after genotyping was not met in 10 of 23 sites from 16 countries with PCR results: Uganda-Amudat, Kenya-Migori, Zanzibar-Micheweni, Uganda-Arua, Sierra Leone-Kailahun, Uganda-Apac, South Sudan-Nuba, Congo-Kinbanda, Rwanda-Rukara, and DRC-Boende (Table 4). Where AS&AQ efficacy PCR-adjusted per site was <90%, the comparator arm was >90% in 3 sites: in Congo-Kinbanda (AL), Uganda-Amudat (AS+SP), and Zanzibar-Micheweni (AL), and not significantly superior to AS&AQ (P > 0.05 for all comparisons, logrank test).

**Table 4:** AS&AQ groups: crude and PCR-adjusted Day 28 efficacy results presented by site and country

Efficacy by Day 28 by country and site *	Number of patients enrolled receiving ASAQ	Crude (not adjusted for reinfection)			PCR-adjusted		
		Efficacy	Lower 95% confidence interval	Upper 95% confidence interval	Efficacy	Lower 95% confidence interval	Upper 95% confidence interval
Angola (all)	166	92.0%	83.0%	96.3%	98.6%	90.5%	99.8%
Angola-Caala	69	91.0%	81.1%	95.9%	98.4%	89.3%	99.8%
Angola-Kuito	97	92.9%	84.9%	96.8%	98.8%	91.7%	99.8%

Burkina Faso (all)	923	96.7%	91.4%	97.5%	NA	NA	NA
Burkina Faso-Bobo-Dilaossou	33	100%	91.3%	100.0%	NA	NA	NA
Burkina Faso-Pouytenga	890	93.5%	91.5%	95.0%	97.3%	95.9%	98.2%

Cameroun-Mendong	110	98.1%	92.5%	99.5%	100.0%	100.0%	100.0%

Congo-Kindamba^#^	101	67.5%	57.0%	75.9%	84.2%	74.8%	90.4%

Gabon-Lambarene	110	87.5%	79.1%	92.7%	94.5%	87.3%	97.7%

Guinea-Dabola	110	94.4%	87.9%	97.4%	99.1%	93.6%	99.9%

Kenya-Migori^#^	200	76.5%	69.1%	82.4%	89.5%	83.4%	93.5%

Madagascar-Tsiroanomandidy	119	96.6%	91.2%	98.7%	99.2%	94.1%	99.9%

Mali (all)	387	74.5%	67.4%	80.4%	98.3%	94.6%	99.5%
Mali-Bancoumana	135	68.5%	59.7%	75.7%	97.5%	92.3%	99.2%
Mali-Bougoula	252	80.6%	75.1%	85.0%	99.2%	96.8%	99.8%

DRC (all)	136	74.5%	62.5%	82.0%	88.3%	76.6%	93.1%
DRC-Boende^#^	90	57.7%	46.5%	67.4%	78.7%	67.6%	86.4%
DRC-Kabalo	46	91.3%	78.5%	96.6%	97.8%	85.6%	99.7%

Rwanda (all)	410	82.4%	75.1%	87.5%	91.6%	85.6%	95.0%
Rwanda-Kicukiro	122	91.6%	85.0%	95.4%	95.8%	90.2%	98.2%
Rwanda-Mashesha	150	86.7%	80.1%	91.2%	95.3%	90.3%	97.7%
Rwanda-Rukara^#^	138	68.8%	60.2%	75.9%	83.8%	76.1%	89.1%

Sénégal (all)	1390	96.1%	91.4%	98.5%	NA	NA	NA
Senegal-Keur Socé	264	94.2%	90.9%	96.7%	99.2%	96.8%	99.8%
Senegal-Djembeye	110	95.3%	91.3%	99.3%	NA	NA	NA
Senegal-Kabrousse	27	100%	89.1%	100.0%	NA	NA	NA
Senegal-Mlomp	883	95.8%	94.4%	97.2%	NA	NA	NA
Senegal-Oussouye	106	95.3%	91.3%	99.3%	NA	NA	NA

Sierra-Leone-kailahun^#^	126	40.5%	31.5%	49.4%	87.5%	78.5%	92.9%

Sudan (all)	214	76.1%	66.8%	83.1%	88.4%	79.8%	93.4%
South Sudan-Nuba^#^	80	63.8%	52.2%	73.2%	84.9%	74.4%	91.4%
Sudan-Malakal	134	88.5%	81.3%	93.0%	91.8%	85.3%	95.5%

Uganda (all)	1283	53.3%	46.5%	59.6%	91.1%	85.1%	94.7%
Uganda-Amudat^#^	106	30.3%	21.3%	39.9%	89.9%	79.2%	95.2%
Uganda-Apac^#^	174	46.5%	38.9%	53.8%	87.2%	80.6%	91.7%
Uganda-Arua^#^	174	49.3%	41.7%	56.6%	88.8%	82.5%	92.9%
Uganda-Jinja	189	79.5%	72.9%	84.6%	94.3%	89.6%	96.9%
Uganda-Kampala	242	83.8%	78.4%	87.9%	95.7%	92.1%	97.7%
Uganda-Tororo	398	30.2%	25.7%	34.8%	91.1%	86.8%	94.0%

Zanzibar (all)	202	75.6%	64.9%	83.3%	91.8%	83.1%	96.2%
Zanzibar-Kivunge	148	73.5%	65.5%	79.9%	95.0%	89.7%	97.6%
Zanzibar-Micheweni^#^	54	77.8%	64.2%	86.7%	88.7%	76.5%	94.7%

*Mean efficacies are given for countries with multiple sites

^# ^Efficacy PCR adjusted <90%

NA: not applicable, no PCR genotyping data for all sites from that country

**Figure 2:**
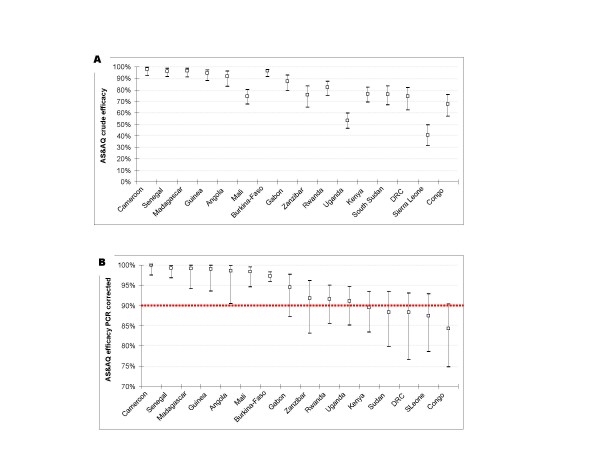
**Crude (A) and PCR-adjusted (B) Day 28 efficacy with ASAQ by country stratified by site (mean and 95% CI)**. Note: The dotted horizontal line in panel B shows the WHO-recommended threshold of efficacy.

The mean efficacy after PCR adjustment by country (Figure 2b) was below 90% in five countries: Kenya (1 site) 89.5% (95% CI 83.4–93.5), South Sudan (2 sites) 88.4% (95% CI 79.8–93.4), Sierra Leone (one site without comparator group) 88.3% (95% CI 76.6–93.1), DRC (two sites) 87.5% (95% CI 78.5–92.9), and Congo (one site) 84.2% (95% CI 74.8–90.4). In all these countries the upper limit of the 95% CI was >90%. Conversely, the lower limit of the 95%CI was above 90% in seven countries (Angola, Burkina-Faso, Cameroon, Guinea, Madagascar, and Senegal).

Out of the five countries where the mean efficacy PCR-adjusted of AS&AQ was <90%, two countries had two studies conducted in a different site: Sudan (Malakal and Nuba) where the mean PCR-adjusted efficacy of AS+SP of both sites was >90% (91.7, 95%CI 85.3–96.4); and DRC (Boende and Kamalo) where the mean PCR-adjusted efficacy of AS+SP was <90% [84.7, 95%CI 76.5–89.1]).

### Secondary efficacy outcomes

#### Parasite clearance

The overall geometric mean of parasite counts on admission (Day 0) was 21,541/μL with wide site/country variations; the geometric means ranged from 3,303/μL in Burkina Faso to 40,492/μL in Dabola, Guinea (p = 0.001). Using multivariate analysis based on randomized comparisons, and controlling by sites, higher parasitaemia was found in younger patients (age as continuous variable) (p = 0.001), and in patients without gametocytes on admission compared to patients with gametocytes (p = 0.001). In the randomized trials, the median parasite clearance time for AS&AQ was Day 2 ranging from Day 1 to Day 7 in 5,853 patients (2.2% did not complete treatment or were regarded as treatment failures). Time to parasite clearance varied from site to site (p = 0.001), and was longer in patients with higher Day 0 parasitaemia (p = 0.001).

**Figure 3:**
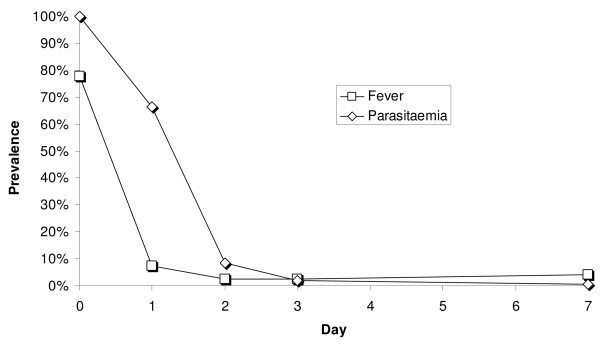
Prevalence rate of patients remaining with parasitaemic or febrile in the first 7 days of follow-up.

The proportion of patients remaining parasitaemic was 66.4% (1634/2462) on Day 1, 8.5% (440/5170) on Day 2, 1.8% (104/5460) on Day 3, and 0.6% (35/5507) on Day 7, including recurrences (Figure 3).

The risk of being parasitaemic on Day 3 in randomized controlled trials was not different between AS&AQ (2.9%, 90/3125) and other ACTs or AS alone (2.6%, 92/2778, p = 0.476, weighted by site). However, patients treated with non-ACT were at a higher risk of remaining parasitaemic on Day 3 (16%, 415/2609, p = 0.001, weighted by site): in AQ groups (19%, 114/611, p = 0.001, weighted by site), in AQ+SP (8%, 103/1256, p = 0.001, weighted by site), and in CQ+SP groups (26%, 184/699, p = 0.001, weighted by site).

The multivariate analysis stratified by site based on randomized comparisons confirmed these results: no difference between AS&AQ and comparators were found using a three-day ACT (p > 0.268 for all comparisons). A significant better parasite clearance was seen with AS&AQ than with non-ACT (the OR for being parasitaemic with AQ, CQ+SP, and AQ+SP was 16.43, 73.11 and 16.71, respectively, p = 0.001 for all comparisons).

#### Fever clearance

All patients included in the trials had fever (axillary temperature ≥ 37.5°C) or a recent history of fever. For the 77.7% (4,614/5,940) AS&AQ patients who were actually febrile on admission, the median fever clearance time was Day 1 (Figure 3). The proportion of patients with fever decreased to 7.4% (372/5,040, 95% CI 6.7%–8.1%) on Day 1, 2.4% (119/4,998, 2.0%–2.8%) on Day 2, and 2.4% (102/4,308, 1.9%–2.8%) on Day 3. Three patients were febrile on Day 21: in one patient from Guinea fever decreased quickly on Day 2 (37.9°C) but remained between 37.5°C and 37.9°C until Day 21, and cleared on Day 28; two other patients who had previously cleared fever became febrile on Day 21 and cleared on Day 28.

Based on randomized comparative studies, the proportion of patients remaining febrile on Day 2 was lower in the AS&AQ groups compared to AS5 (0.8%, 2/251 vs. 4.0%, 10/252, p = 0.020), AS+SP (1.4%, 7/487 vs. 4.1%, 20/488, p = 0.011), CQ+SP (1.5%, 7/745 vs. 4.7%, 33/697, p = 0.001), AL (2.8%, 33/1,178 vs. 6.3%, 63/1,000, p = 0.001). No difference was detected between AS+AQ and AQ alone, DP, or AQ+SP and between the non-fixed and fixed ASAQ products (p > 0.147 for all comparisons).

### Risks of failure in randomized comparisons

**Figure 4:**
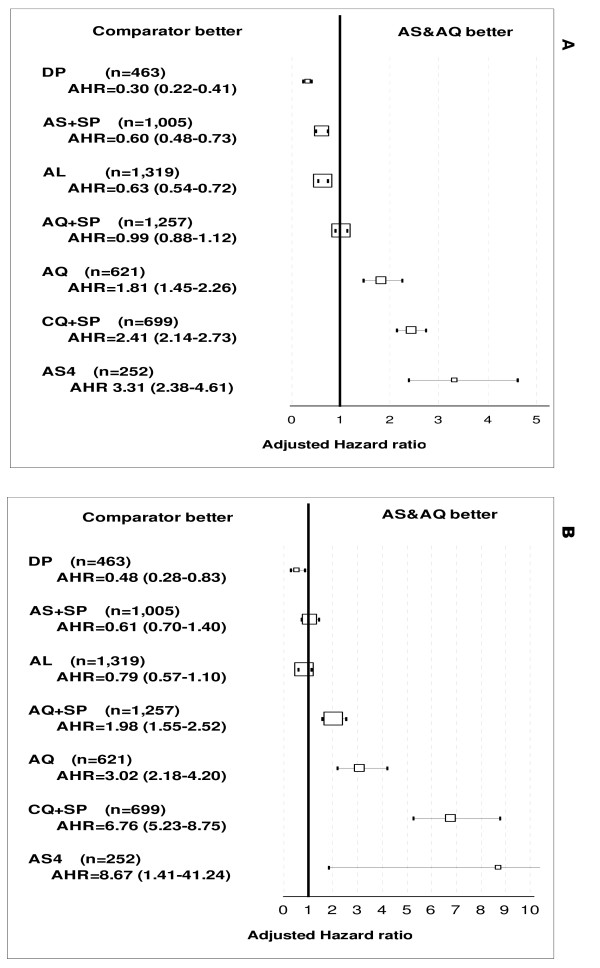
**Overall risks of failure of artesunate-amodiaquine by comparator: (A) crude, (B) PCR-adjusted Day 28 outcome.** Note: The forest plot represents the risk of failure of artesunate amodiaquine versus comparators in randomized comparative studies. Results were stratified by site. The size of boxes is proportional to the number of patients included. The square represents the adjusted hazard ratio and 95% CI.

Recurrence. The randomized comparative clinical trial conducted in Burkina Faso did not detect any difference in crude efficacy (PCR not adjusted) between the loose (AS+AQ) and the fixed-dose (ASAQ) combinations (p = 0.510). Based on randomized comparative studies with AS&AQ (N = 4,896) compared to other anti-malarial treatments (N = 5,713) and using multivariate analysis stratified by site (Figure 4a), patients treated with DP, AL, and AS+SP were at lower risk of failure (p = 0.001, for all comparisons) compared to AS&AQ, while patients treated with AQ alone, AS5 and CQ+SP were at a higher risk (p = 0.001, for all comparisons). The risk of failure was not different between AS+AQ and AQ+SP (p = 0.812).

Recrudescence. In the non-AS&AQ comparator arms, genotyping results were available for all studies analysed (N = 21). In Burkina Faso, no difference was detected between the fixed-dose 95.0% (95% CI 92.7–97.3) and the non-fixed combination 95.7% (95% CI 93.7–97.7) (p = 0.645). Based on comparative randomized trials, and using multivariate analysis stratified by site, the risk of failure compared with AS&AQ was (i) lower with DP (p = 0.001); (ii) higher with AQ+SP, AQ alone, AS5, and CQ+SP (p = 0.001 for all comparisons); (iii) not different as compared with AS+SP and AL (p = 0.346; p = 0.158, respectively) (Figure 4b).

### Predictors of failure

Recurrence. In AS&AQ groups using multivariate analysis stratified by site and controlling for potential independent factors (age, parasitaemia, and gametocyte on admission), younger children (age in continuous in terms of per 1 year increase of age AHR = 0.93, 95% CI 0.90–0.97, p = 0.001), and anaemic compared to non-anaemic patients were at a higher risk of failure (AHR = 1.17, 95% CI 1.02–1.35, p = 0.022). Likewise, when reinfections (PCR confirmed) were included for analysis, younger (AHR = 0.96, 95% CI 0.92–0.99, p = 0.023) and anaemic patients (AHR = 1.21, 95% CI 1.04–1.42, p = 0.014) were at higher risks.

Recrudescence. The median time to recrudescence (PCR confirmed) with AS&AQ was Day 21. Using similar analysis as previously, younger patients were also at higher risk for recrudescence (AHR = 0.88, 95% CI 0.81–0.95, p = 0.001), and no other independent factor was detected.

### Gametocytaemia

**Figure 5:**
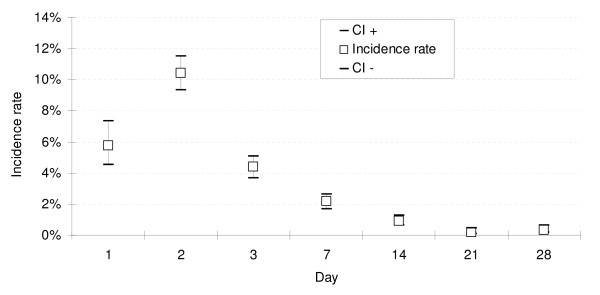
**Incidence rate of gametocyte appearance in AS&AQ groups by Day in patients without gametocyte on admission**.

On admission. In AS&AQ groups, the prevalence rate of gametocytaemia on admission was 12.9% (95% CI 5.4%–20.5%), ranging from 0% in Zanzibar-Micheweni and Cameroon-Mendong, to 51.7% in Uganda-Apac. Using multivariate analysis and controlling for site, younger patients (p = 0.001) and patients with lower parasitaemia (p = 0.001) were at a higher risk for gametocytaemia on admission. The overall cumulative incidence probability of gametocyte presence on Day 28 was 31.4% (95% CI 22.7–39.7). The peak of gametocyte prevalence was on Day 2 (19.3%, 677/3,516). The gametocyte carriage rate was 71 PGW per 1,000 weeks of follow-up, and the mean duration per patient was 14.5 (SD ± 11.7) days. In patients who did not have gametocytes on admission, the cumulative probability was 20.4% (95% CI 18.3–22.5), and the gametocyte carriage was 36/1,000 PGW. The mean duration was 6.2 (SD ± 2.5) days, and the maximum incidence rate was reached on Day 2: 10.4% (95% CI 9.3–11.5) when 48% of the cases occurred (Figure 5).

**Figure 6:**
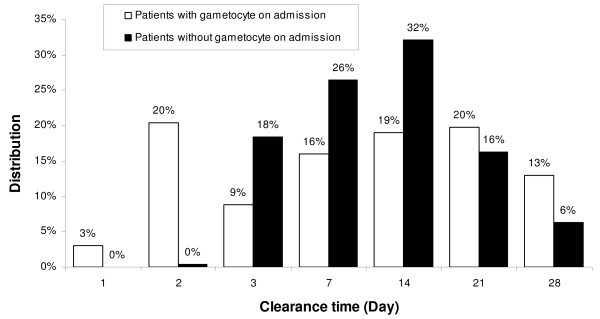
**Gametocyte clearance time distribution in AS&AQ groups in patients with and without gametocyte on admission**.

Gametocyte clearance. The overall median clearance time was Day 14 in patients treated with AS&AQ, and varied widely by site (ranging from Day 1 to 28). Clearance time could not be calculated for 7.7% (46/594) of the patients with gametocytes on admission who had been lost to follow-up or censored due to failure, leaving 548 patients for the analyses on Day 28 (all having cleared their gametocytes by then). There was no difference in clearance time between patients who had gametocytes on admission and those who developed gametocytaemia post-admission (p = 0.378). However, while the peak time to clearance was Day 14 for the patients who did not have gametocyte on admission (32%), gametocyte clearance for those who had gametocytes on admission was almost evenly distributed throughout treatment and follow-up (20% on Day 2 and Day 21) (Figure 6).

Results by drug treatment. Using survival analysis to examine the cumulative probability of gametocyte appearance in patients who did not have gametocytes on admission, and measuring levels of carriage expressed in PGW by site, different profiles were obtained depending on the anti-malarial used. No difference in gametocyte appearance was detected between the loose and fixed AS&AQ combinations (AHR = 1.07, p = 0.587).

**Figure 7:**
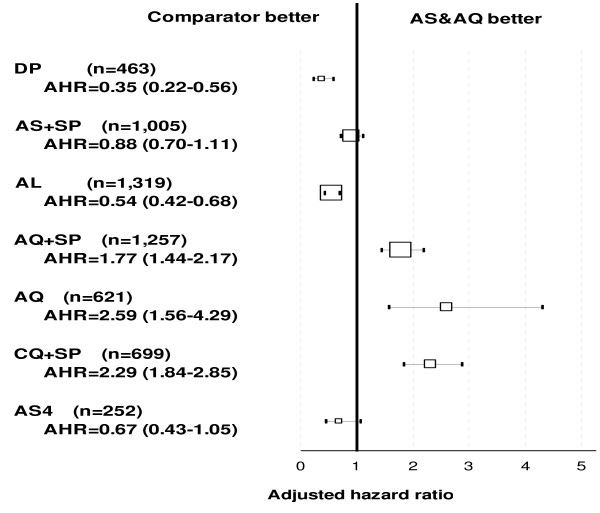
**Overall risks of gametocyte appearance in artesunate-amodiaquine groups by drug comparator**. Note: The forest plot represents the risk of failure of artesunate amodiaquine versus comparators in randomized comparative studies. Results were stratified by site. The size of boxes is proportional to the number of patients included. The square represents the adjusted hazard ratio and 95% CI.

Overall, using multivariate analysis based on randomized comparative studies and stratified by site (Figure 7), the risk of gametocyte appearance post-admission compared to AS&AQ groups was higher with AQ (AHR = 2.59, p = 0.001), CQ+SP (AHR = 2.29, p = 0.001), and AQ+SP (AHR = 1.77, p = 0.001); lower with AL (AHR = 0.57, p = 0.001) and DP (AHR = 0.39, p = 0.001); and not different with AS+SP (AHR = 0.88, p = 0.288).

Gametocyte carriage rate in patients without gametocyte on admission in randomized comparative trials.

**Figure 8:**
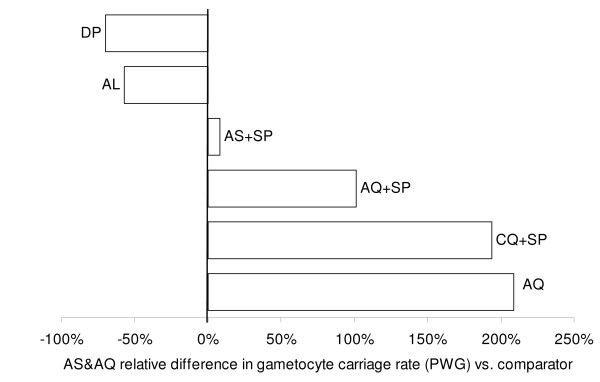
**Relative difference in gametocyte carriage rate (person-gametocyte-week, PGW) in artesunate amodiaquine groups and comparators in randomized comparative studies**.

No difference was detected between the loose and the fixed AS&AQ combinations in Burkina Faso (p = 0.824). The overall carriage rate was 57% shorter with AL (13/1000 PGW) compared with AS&AQ (27/1000 PGW, RR_MH _[Mantel-Haenszel rate ratio] = 0.48, 95% CI 0.31–0.63, p = 0.001)(Figure 8). However, results by site showed that AL was only superior to AS+AQ (RR = 0.05, 95% CI 0.01–0.14, p = 0.001) in one site in Uganda (out of 3 Ugandan sites, and not in the other sites). Compared with DP groups in Rwanda, no difference in gametocyte carriage was detected compared to AS+AQ (p = 0.817). As a result, the overall carriage rate was 70% shorter in DP groups (RR_MH _= 0.25, 95% CI 0.11–0.41, p = 0.001, weighted by site). However, the carriage rate was only significantly lower in DP compared to AS&AQ groups in one Ugandan site (RR = 0.12, 95% CI 0.06–0.26, p = 0.001). Conversely, AS+SP (57/1000 PGW) increased the overall gametocyte carriage rate by 8% versus AS+AQ, but not significantly so (53/1000 PGW, RR_MH _= 1.15, 95% CI 0.78–1.69, p = 0.514, weighted by site).

Treating patients with AQ+SP significantly increased the overall carriage rate by 101% from 36/1000 to 72/1000 PGW vs. AS+AQ (RR_MH _= 1.95, 95% CI 1.56–2.40, p = 0.001, weighted by site). Using CQ+SP (157/1000 PGW) also increased the overall carriage rate by 194% compared to AS+AQ (53/1000 PGW, RR_MH _= 3.00, 95% CI 3.36–3.71, p = 0.001, weighted by site). Overall gametocyte carriage with AQ alone (45/1000 PGW) was also superior by 209% compared to AS+AQ combination (15/1000 PGW, RR_MH _= 3.34, 95% CI 1.97–5.71, p = 0.001, weighted by site).

### Anaemia

The prevalence of anaemia at baseline was 49.4% (5031/10194), ranging from low levels (2.9%, 4/140) in Senegal-Mlomp, to high levels (86.7%, 91/105) in Zanzibar-Micheweni.

Predictors of anaemia. Using multivariate analysis and controlling for sites, patients in the AS&AQ group who had gametocyte on admission were at a higher risk for anaemia (AOR = 1.56, 95% CI 1.23–1.98, p = 0.001), as were younger children (AOR = 0.66, 95% CI 0.62–0.70, p = 0.001).

Effects of AS&AQ treatment on anaemia in randomized trials. In patients treated with AS&AQ and followed until Day 28, 62% (1,764/2,863) were anaemic at enrolment. Of these, 25% (709/2,863) had mild (8–9.9 g/dL, grade 2), 26% (745/2,863) moderate (5–7.9 g/dL, grade 3) and 11% (310/2,863) severe anaemia (<5 g/dL, grade 4). By Day 28, 38% (678/1,764) of the patients had recovered, 62% (1,086/1,764) remained anaemic, and 9% (104/1,099) who were not anaemic on admission became anaemic (of which 9% [9/104] had severe anaemia). For 88% (274/310) of the patients who had severe anaemia on admission, the severity of anaemia was reduced. By Day 28, anaemia in these 274 patients became moderate in 273 patients (99%) and mild in 1 patient (<1, 1/274). Severe anaemia remained unchanged in 12% (36/310). Overall, less than 1% (9/2553) of the patients developed severe anaemia post treatment. Paired analysis of Day 0 and Day 7 showed a significant transient decline in Hgb count (-28 g/dL, SD 1.17, -3%, 95% CI -5 to -1, p = 0.001) followed by a significant increase on Day 28 (+1.16 g/dL, SD 1.63, +13%, 95% CI 12 – 15, p = 0.001) compared to Day 0.

Results by drug treatment (Table 5).

**Table 5:** Haemoglobin and haematocrit values and changes between Day 0–14, and Day 0–28 (AS&AQ and comparators)

**Haemoglobin**		N	Day 0		Day 7		Paired relative difference			Paired difference		t-value	P-value
			mean	sd	Mean	sd	%	lower 95%CI	upper 95%CI	mean	sd		
Ndiaye	ASAQ	766	10.1	2.29	9.6	2.02	-5%	-3%	-7%	-0.50	1.24	-11.07	0.000
	AL	482	9.9	2.26	9.7	1.92	-2%	1%	-4%	-0.18	1.40	-2.89	0.004
Epicentre	AS+AQ	249	10.3	1.76	9.8	1.74	-4%	-1%	-7%	-0.42	1.19	-5.50	0.000
	AS+SP	248	10.2	1.88	9.7	1.66	-5%	-2%	-8%	-0.51	1.20	-6.71	0.000

**Haematocrit**		N	Day 0		Day 7		Paired relative difference			Paired difference		t-value	P-value
			mean	sd	Mean	sd	%	lower 95%CI	upper 95%CI	mean	sd		

Sirima	AS+AQ	136	25.5	4.93	27.4	4.57	7%	4%	10%	1.87	3.75	5.83	0.000
	ASAQ	149	25.8	4.52	27.5	4.25	6%	4%	9%	1.64	3.12	6.40	0.000

**Haematocrit**		N	Day 0		Day 14		Paired relative difference			Paired difference		t-value	P-value
			mean	sd	Mean	sd	%	lower 95%CI	upper 95%CI	mean	sd		

Karema	AQ+SP	251	31.5	5.35	34.49	3.70	9%	8%	11%	2.95	4.95	9.44	0.000
	AS+AQ	247	31.0	5.49	33.99	3.66	10%	8%	11%	3.02	4.88	9.72	0.000
	DP	247	31.5	4.91	33.43	3.62	6%	4%	8%	1.90	5.24	5.70	0.000

**Haemoglobin**		N	Day 0		Day 28		Paired relative difference			Paired difference		t-value	P-value
			mean	sd	mean	sd	%	lower 95%CI	upper 95%CI	mean	sd		

Ndiaye	ASAQ	762	10.1	2.31	11.04	1.76	9%	7%	11%	0.96	1.74	15.48	0.000
	AL	471	9.96	2.26	10.93	1.59	10%	7%	12%	0.97	1.69	12.52	0.000
Epicentre	AS+AQ	798	9.60	1.93	10.68	1.59	11%	9%	13%	1.08	1.68	18.18	0.000
	AS+SP	801	9.54	1.94	10.64	1.50	12%	10%	13%	1.10	1.66	18.77	0.000

**Haematocrit**		N	Day 0		Day 28		Paired relative difference			Paired difference		t-value	P-value
			mean	sd	mean	sd	%	lower 95%CI	upper 95%CI	mean	Sd		

Sirima	AS+AQ	259	26.7	6.00	31.38	4.50	18%	16%	20%	4.70	5.85	12.92	0.000
	ASAQ	258	26.7	5.59	31.33	5.02	17%	15%	20%	4.66	5.78	12.94	0.000

Note. SD: standard deviation; CI: confidence interval

In randomized comparative trials, in patients followed up until Day 28, 46% (353/762) in the AS&AQ groups, and 32% (246/471) of the patients in the AL groups were anaemic on admission (p = 0.044). On Day 28, in AS&AQ groups, 54% (190/353) of the patients recovered, 46% (163/353) remained anaemic, and 10% (39/409) who were not anaemic on admission became anaemic (none had severe anaemia <5 g/dL). On Day 28, in the AL groups, 56% (138/246) recovered from anaemia, 44% (108/246) remained anaemic, and 8% (19/225) became anaemic (none had severe anaemia). There was no difference in proportions of patients recovering, remaining, or becoming anaemic between these groups. A paired analysis of Days 0 and 7 showed a significant transient decline in both groups in Hgb count (-5%, 95% CI -3 to -7, p = 0.001; -2%, 95% CI -4 to -1, p = 0.004, respectively). The mean paired difference decrease was greater in AS&AQ compared to AL groups (p = 0.001). By Day 28, the relative mean paired difference increased significantly in both groups with no difference between the two groups (+9%, 95% CI +7 to +11, p = 0.001; +10%, 95% CI +7 to +12, p = 0.001, respectively; p = 0.917).

Similar paired analysis results of Day 0 and Day 14 (Table 5) were observed in randomized comparative trials with AQ+SP and DP, as well between Days 0 and 28 in patients treated with AS&AQ (p = 0.001 for all comparisons). No significant differences were observed in variations of Hgb between treatment arms, except in Rwanda [13] where the recovery in relative mean paired Hct difference in the AS+AQ group (+10%, 95% CI 8 to 11) was significantly higher than in the DP group (6%, 95% CI 4 to 8) (p = 0.021).

In trials comparing AS+SP and AS+AQ (Table 5), a similar transient decrease was observed in various settings on Day 7 until recovery on Day 28 (+13% for both comparisons) without any variation difference comparing the drugs (Day 7: p = 0.372, Day 28: p = 0.772).

In Uganda, there was no significant difference in haemoglobin levels in the AS+AQ group on Day 14 compared to Day 0 (-0.4%, p = 0.551) whereas in the comparative AQ+SP group, a significant relative mean paired increase was detected (+1.3%, p = 0.016). The mean paired difference between the two groups being significant (p = 0.039). On Day 28 the variation was no longer different between the two groups (+15%, +16%, respectively, p = 0.406).

In Burkina Faso [5] no difference was detected between the loose and the fixed AS&AQ combinations between Days 0 and 28 (+17%, +18%, respectively, p = 0.946).

## Discussion

This individual patient analysis has pooled data from 26 drug trials in a majority of paediatric malaria cases in sub-Saharan Africa identified through a systematic search and has focused on efficacy; safety will be reported separately. The trials reported herein were conducted between July 1999 and December 2006; thus, this analysis provide recent information on the current situation. Both absolute and comparative efficacy results varied between crude and PCR-adjusted results (i.e. whether reinfections are counted or discounted in the analysis).

The WHO recommends using treatments that are at least 90% effective after discounting reinfections [2]. Overall, AS&AQ had an efficacy of ~94% after excluding reinfections by PCR. However, 10 sites in eight countries (out of 28 sites in 16 countries) failed to meet the WHO, Day 28, PCR-adjusted cut-off of >90% efficacy. These sites were in Congo, DRC, Kenya, Sierra Leone, South Sudan, Rwanda, Uganda, and Zanzibar. However, at other sites in some of these countries (DRC, Rwanda, South Sudan, Uganda, and Zanzibar), the PCR-adjusted efficacy exceeded 90%. Moreover, the PCR-adjusted efficacy in the comparator arm was >90% only in three sites (Kindamba-Congo, Amudat-Uganda, Micheweni-Zanzibar), and was not significantly superior to AS&AQ.

The definition of recrudescent failure was strict since recurrent parasitaemia that could not be successfully genotyped by PCR (indeterminate case) was considered conservatively as a recrudescent failure. This was done in order to prevent from introducing overestimation bias in assessing AS&AQ efficacy levels, in comparison to other attrition methods by modified intent-to-treat analysis that would increase the level of efficacy by excluding the PCR indeterminate cases from the analysis. Compared to other treatments, AS&AQ was either superior to non-ACTs or not different from AS+SP and AL but inferior to DP.

As expected, the AS&AQ crude efficacy, which counts reinfections as failures, was much lower, ~78% (with wide inter-country variability), than was the PCR-adjusted efficacy (~94%). During the 28 days of follow-up, the quotients of failure in the AS&AQ groups were the greatest on Day 21 and Day 28, in contrast with the other forms of ACT, for which the peak was reached on Day 28. When the risk of a reinfection is high in areas of intense transmission, treatment with longer post-treatment protection (AL, AS+SP, DP) fared better than AS&AQ. This probably reflects the relatively shorter residence time of AQ in the human body such that concentrations of the active metabolite, monodesethyl-amodiaquine, might be lower or absent when a reinfection occurs compared to other partner drugs combined to artemisinin derivatives. In the crude analysis of efficacy, AS&AQ was inferior to DP, AL and AS+SP.

Whether a short or a longer residence time for a drug is preferable is a matter of debate. Operationally, post-treatment protection against reinfection is a positive feature as it minimizes the number of treatments needed by the individual, the frequency of contacts with health providers, the risk of cumulative toxicity, and the costs (direct and indirect) incurred by households and health systems. Conversely, persisting concentrations of low drug levels may be insufficient to inhibit the replication of parasites arising from a new infection and potentially select for the parasites that can tolerate those levels. Furthermore, results depend on the study design and the duration of follow-up. It might be difficult to judge the operational implications of reinfections and re-treatment based on studies of treatment of single episodes of malaria; prospective cohort studies are best suited to assess the consequences of repeat treatments.

Based on the Day 28 efficacy results of these studies, AS&AQ would be suitable according to WHO standards as a potential alternative treatment for *P. falciparum *malaria in Angola, Burkina Faso, and Mali, where the current first line is AL. AS&AQ satisfied the criteria for continued use in some of the countries where is the current first-line treatment (Cameroon, Guinea, Madagascar, Gabon, Senegal, and Zanzibar), but AS&AQ would not qualify in some sites in Sierra Leone, Congo, DRC, North and South Sudan, Rwanda, Kenya, Uganda. However, where the AS&AQ efficacy PCR-adjusted was <90%, and the one of the comparators was >90%, the comparator groups were never significantly superior to AS&AQ whether in Congo (AL), Rwanda (DP), or Uganda (AS+SP).

This multi-centre analysis provides also interesting information on malaria and response to treatment. It confirms that children under 5 years of age are particularly vulnerable, as they are more likely to have on presentation higher baseline parasitaemia, be anaemic and carry gametocytes, and have a higher risk of failure compared to older children for all treatments evaluated, consistent with a lack of malaria-acquired immunity [44].

Being young and anaemic increases the risk for antimalarial treatment to fail to clear parasites and to be reinfected after clearing the current infection, suggesting a relationship between anaemia and transmission intensity, and between anaemia and susceptibility to infection. Conversely, young age alone predicts recrudescence after initial clearance.

Young children are a major reservoir of gametocytes and hence the engine of malaria transmission. Gametocyte carriage is highest when asexual parasitaemia is low.

Fever clearance was fast with AS&AQ, similar to other forms of ACT and AQ+SP, but faster than AL, and other non-ACT. Parasite clearance was fast with AS&AQ, generally faster than non-ACT and similar to other forms of ACT.

The presence of gametocytes on admission ranged from 0 to ~50% across the studies, and was related to young age and low asexual parasitaemia. The cumulative risk of gametocytes appearing post-treatment was ~20% with 36 PGW carriage per 1000 weeks of follow-up. The gametocyte clearance time in AS&AQ groups was the same (median 14 days) whether patients presented with gametocytes or developed gametocytaemia thereafter, but the peak distributions of time to clearance were Day 2 and Day 14, respectively. Compared to AS&AQ, the risk of appearance of gametocytes was higher and the carriage duration was longer with the non-ACTs and AS+SP, but lower with DP and AL in one Ugandan site, consistent with their better efficacy against asexual parasites.

Endemic countries are faced with the challenge of identifying the treatment(s) best adapted to their needs. To inform decisions, both locally generated data and more general information are needed. Systematic reviews and meta-analyses are useful to summarize evidence and assist policy makers. Pooling individual patient data offers advantages over aggregate patient data meta-analysis because it allows standardizing patient attrition and analyses. Each study can then be re-analysed based on common criteria for efficacy and safety and different drug regimens can be combined and compared. Data can also be combined and analysed together while stratifying by site. Efficacy analyses can be done on modified intent-to-treat basis of all randomized patients and use Kaplan-Meier product-limit estimates of time to event. This is now the preferred analytical method for anti-malarial drug efficacy trials [45].

Individual studies are not usually designed and, therefore, not powered to detect differences in a variety of secondary outcomes (e.g. gametocyte carriage, parasite or fever clearance time). Results of this analysis of individual patient data were presented using similar methods to that used for a conventional meta-analysis of trials (for instance in Cochrane's review) with graphical representation of risks, recommended for communicating in medical research [46]. Compared to a meta-analysis from published studies, combining and standardizing these data at patient level increases statistical power by facilitating analytical practice (sub-group and multivariate analyses stratified by site) despite significant heterogeneity between trials. It also enables standardized estimates of drug efficacy across different studies, and the identification of at-risk groups to help target public health strategies.

However, this individual patient multi-centre analysis is not without limitations. First, the analysis included only half, 25 of the 46 trials that met criteria of quality for inclusion. It also has excluded additional trials published past August 2008, due to the time needed to adequately harmonize published data, obtain additional reported data and conduct the analyses. This might be a source of bias. The Worldwide Antimalarial Resistance Network (WARN) [47,48] intends to create a living database, which might become the basis for updated assessments of drug efficacy. Second, these results apply primarily to children under five years of age (75% of the patients enrolled) and less to older children or adults. However, young children are indeed those at higher risk and are the primary target of malaria interventions. Finally, this analysis showed heterogeneity of study results both across and within countries, a finding that illustrates the challenges faced when making drug policy decisions. Differences in efficacy between sites might have resulted from the variability in the composition of the study drug, as well as PCR methods that have been used according to sites facilities.

A Cochrane systematic review and meta-analysis which includes AS&AQ [49] has just been published with consistent results.

At a bare minimum, malaria control programmes need up-to-date, dynamic, and comparative data on anti-malarial drug efficacy and safety in order to recommend optimal drug treatments for their countries. Prospective multi-centre analysis could be a key element for deciding drug policy at national and regional levels.

## Competing interests

The authors declare that they have no competing interests.

## Authors' contributions

JZ and PO designed the analyses and were the primary writers of the manuscript. JZ pooled the data and conducted the analysis. All the principal investigators from the AS&AQ Individual Patient Data (IPD) study group contributed data and participated in the writing or approved the manuscript. We thank the collaborating centres for sharing their data.
